# Deconvolution of whole blood transcriptomics identifies changes in immune cell composition in patients with systemic lupus erythematosus (SLE) treated with mycophenolate mofetil

**DOI:** 10.1186/s13075-023-03089-5

**Published:** 2023-06-30

**Authors:** Mumina Akthar, Nisha Nair, Lucy M. Carter, Edward M. Vital, Emily Sutton, Neil McHugh, British Isles Lupus Assessment Group Biologics Register (BILAG BR) Consortium, Patrick Gordon, Steven Young-Min, Robert Stevens, Athiveer Prabu, Mike Batley, Nagui Gendi, Bhaskar Dasgupta, Munther Khamashta, Peter Hewins, Richard J. Stratton, Antoni Chan, Denise De Lord, Jon King, Shirish Dubey, Edmond O’Riordan, Shireen Shaffu, Cathy Laversuch, Thomas P. Sheeran, Erin Vermaak, Nicola Erb, Debasish Pyne, Rachel Jeffrey, Hazem Youssef, Wahab Al-Allaf, Marian Regan, Arvind Kaul, MASTERPLANS Consortium, Katherine Payne, Mark Lunt, Niels Peek, Nophar Geifman, Sean Gavan, Gillian Armitt, Patrick Doherty, Jennifer Prattley, Narges Azadbakht, Angela Papazian, Helen Le Sueur, Carmen Farrelly, Clare Richardson, Zunnaira Shabbir, Lauren Hewitt, Caroline Gordon, Stephen Young, David Jayne, Vern Farewell, Li Su, Matthew Pickering, Elizabeth Lightstone, Alyssa Gilmore, Marina Botto, Timothy Vyse, David Lester Morris, David D’Cruz, Miriam Wittmann, Paul Emery, Michael Beresford, Christian Hedrich, Angela Midgley, Jenna Gritzfeld, Michael Ehrenstein, David Isenberg, Mariea Parvaz, Jane Dunnage, Jane Batchelor, Elaine Holland, Pauline Upsall, Ian N. Bruce, John A. Reynolds

**Affiliations:** 1 Rheumatology Department, Sandwell and West Birmingham NHS Trust, Birmingham, UK; 2 Centre for Genetics and Genomics Versus Arthritis, Centre for Musculoskeletal Research, Manchester Academic Health Science Centre, The University of Manchester, Manchester, UK; 3 Leeds Institute of Rheumatic and Musculoskeletal Medicine, University of Leeds, Leeds, UK; 4 NIHR Leeds Biomedical Research Centre, Leeds Teaching Hospitals NHS Trust, Leeds, UK; 5 Centre for Epidemiology Versus Arthritis, Division of Musculoskeletal & Dermatological Sciences, The University of Manchester, Manchester, UK; 6 Department of Pharmacy and Pharmacology, University of Bath, Bath, UK; 7 NIHR Manchester Biomedical Research Centre, Manchester University Hospitals NHS Foundation Trust, Manchester Academic Health Science Centre, Manchester, UK; 8 Rheumatology Research Group, Institute of Inflammation and Ageing, College of Medical and Dental Sciences, University of Birmingham, Birmingham, UK

**Keywords:** Systemic lupus erythematosus, Transcriptomics, Deconvolution, Mycophenolate mofetil

## Abstract

**Background:**

Systemic lupus erythematosus (SLE) is a clinically and biologically heterogeneous autoimmune disease. We explored whether the deconvolution of whole blood transcriptomic data could identify differences in predicted immune cell frequency between active SLE patients, and whether these differences are associated with clinical features and/or medication use.

**Methods:**

Patients with active SLE (BILAG-2004 Index) enrolled in the BILAG-Biologics Registry (BILAG-BR), prior to change in therapy, were studied as part of the MASTERPLANS Stratified Medicine consortium. Whole blood RNA-sequencing (RNA-seq) was conducted at enrolment into the registry. Data were deconvoluted using CIBERSORTx. Predicted immune cell frequencies were compared between active and inactive disease in the nine BILAG-2004 domains and according to immunosuppressant use (current and past).

**Results:**

Predicted cell frequency varied between 109 patients. Patients currently, or previously, exposed to mycophenolate mofetil (MMF) had fewer inactivated macrophages (0.435% vs 1.391%, *p* = 0.001), naïve CD4 T cells (0.961% vs 2.251%, *p* = 0.002), and regulatory T cells (1.858% vs 3.574%, *p* = 0.007), as well as a higher proportion of memory activated CD4 T cells (1.826% vs 1.113%, *p* = 0.015), compared to patients never exposed to MMF. These differences remained statistically significant after adjusting for age, gender, ethnicity, disease duration, renal disease, and corticosteroid use. There were 2607 differentially expressed genes (DEGs) in patients exposed to MMF with over-representation of pathways relating to eosinophil function and erythrocyte development and function. Within CD4 + T cells, there were fewer predicted DEGs related to MMF exposure. No significant differences were observed for the other conventional immunosuppressants nor between patients according disease activity in any of the nine organ domains.

**Conclusion:**

MMF has a significant and persisting effect on the whole blood transcriptomic signature in patients with SLE. This highlights the need to adequately adjust for background medication use in future studies using whole blood transcriptomics.

**Supplementary Information:**

The online version contains supplementary material available at 10.1186/s13075-023-03089-5.

## Introduction

Systemic lupus erythematosus (SLE) is an autoimmune, immune complex-mediated disease associated with systemic inflammation and the production of autoantibodies. The marked heterogeneous nature of SLE, both clinically and biologically, has continued to impose challenges in terms of understanding the pathogenesis of SLE, in drug development and in clinical care [1, 2].

Transcriptomic profiling has the potential to accelerate our understanding of the biology of SLE. It may contribute to improving the classification of SLE, allow exploration of disease mechanisms, and help to identify novel treatment targets or personalised approaches to treatment [2]. Previous transcriptomic studies in SLE have demonstrated the contribution of IFNβ and IFNγ (in addition to IFNα) to SLE pathogenesis and the presence of distinct gene signatures for susceptibility, disease activity, and severity [2, 3]. However, the marked clinical and molecular heterogeneity of SLE can make transcriptomic analysis at either the transcript or gene level challenging and may lead to discrepancies between studies. Consideration of the cell proportions within bulk RNA sequencing (RNA-seq) data may yield important insights and improve the statistical power of transcriptomic analyses.

CIBERSORTx is a deconvolution-based computational method which uses support vector regression in combination with the knowledge of expression profiles in a signature matrix to accurately estimate the relative immune proportions of cells from bulk tissue transcriptomes [4, 5]. CIBERSORTx has been used to estimate immune cell proportions in peripheral blood in several conditions including ischaemic stroke [6], schizophrenia [7], and liver cirrhosis [8]. The data obtained from the CIBERSORTx pipeline has been validated against clinical laboratory measurements and/or flow cytometry data [7, 8]. Recently, it has been reported that immune cell scoring systems, based on CIBERSORTx data, can predict prognosis in patients with myelodysplastic syndromes [9].

Deconvolution of microarray data has been used in studies comparing patients with SLE to healthy controls. Patients with SLE had increased monocytes and fewer NK cells in the peripheral blood compared to healthy controls [10]. Similarly, deconvolution of microarray data from renal tissue identified differences in cell proportions between glomeruli from patients with lupus nephritis compared to healthy donors with increased monocytes, macrophages, and activated natural killer (NK) cells and fewer memory B cells and T follicular helper cells in LN tissue [11].

In this study, we aimed to use CIBERSORTx to determine whether deconvolution of whole blood RNA-seq data could identify differences in predicted immune cell proportions between active SLE patients and whether these differences are associated with the clinical phenotype of patients or with concomitant medication use.

## Methods

### Study cohort

Patients with active SLE who fulfilled the 1997 Updated American College of Rheumatology (ACR) classification criteria for SLE [12] or the SLICC 2012 criteria [13] were registered with the BILAG-Biologics Registry (BILAG-BR). Ethical approval was granted by North West Greater Manchester West Research Ethics Committee (09/H1014/64) and the local Research and Development departments at participant sites. This cohort formed a key component of the UK Medical Research Council (MRC) Precision Medicine Consortium ‘Maximising SLE Therapeutic Potential by the Application of Novel and Stratified Approaches’ (MASTERPLANS). Disease activity was quantified using the BILAG-2004 index [14]. Blood samples were collected at enrolment into the registry for whole blood RNA-seq and autoantibody profiles were measured in a central laboratory. Routine biochemical, haematological, and serological parameters were measured locally.

### Whole blood bulk RNA sequencing (RNA-seq)

RNA was extracted from PAXgene tubes and RNA integrity was analysed using the Agilent 2100 Bioanalyzer. Complementary DNA synthesis was performed using the Illumina® TruSeq RNA Sample Preparation Kit (Illumina), and pooled cDNA libraries were sequenced using the HiSeq 2000 Illumina® platform (Illumina). Quality assessment was performed using FastQC (http://www.bioinformatics.babraham.ac.uk/projects/fastqc/), quality filtering with Trimmomatic [15], read mapping to hg38, and counting into genes with STAR [16] using annotation from GENCODE v24 (http://www.gencodegenes.org/). Fragments per kilobase million (FPKM) were determined for downstream analysis using *RNAAgeCalc* [17]. Differential gene expression was determined using *DESeq2* [18]. Gene set enrichment analysis (GSEA) was performed using *ClusterProfiler* using the genome wide annotation for human (*org.Hs.eg.db*). Gene ontology (GO) analysis was conducted using GOnet [19]. Hierarchical clustering with bootstrapping was performed using *factoextra.*

### Deconvolution of gene expression profiles

Bulk RNA-seq deconvolution and cell type estimation was performed using CIBERSORTx and the LM22 leukocyte gene signature matrix [4, 5]. LM22 comprises 547 genes, derived from human microarray data, to distinguish 22 human immune cell types [5]. The following specifications were used: B-mode batch correction, quantile normalisation disabled, 100 per mutations and relative-mode. For some analyses, absolute mode was used. For further details, see Supplementary methods. For high resolution mode, only protein-coding transcripts were used.

The relative cell proportions obtained from CIBERSORTx were compared between set groups using non-parametric tests (Mann–Whitney *U* test with Benjamini–Hochberg correction or Kruskal–Wallis test with Dunn’s correction where appropriate) with the FDR set at 0.05. In the adjusted models, cell frequency was converted into quartiles and ordered logistic regression models to determine the odds ratio for moving between quartiles. Data were analysed using IBM® SPSS® Statistics 26, STATA v.16.0 and R v4.0.3.

## Results

### Patient characteristics

Whole blood transcriptomic data were available for 110 patients; one of whom had incomplete clinical data and was therefore excluded from analyses. The cohort included 104/109 (95.4%) females, with a median (IQR) age and disease duration of 38 (29–49) and 10 (6.5–16.5) years respectively. Patients had high disease activity with a median SLEDAI score of 8 (4–14) (Table 1).

**Table 1:** Description of the study population

Patient characteristics, total (*n* = 109)	No (%)/median (IQR)
Age, years (*n* = 98)	38 (29–49)
Sex	
Female	104 (95.4%)
Male	5 (4.6%)
Ethnicity	
White	61 (56%)
Black	14 (12.8%)
South Asian	20 (18.3%)
Other	11 (10.1%)
Not Specified	3 (2.8%)
SLE duration, years	10 (6.5–16.5)
1997 ACR criteria	
Malar rash	60 (55%)
Discoid rash	15 (13.8%)
Photosensitivity	59 (54.1%)
Oral ulcers	68 (62.4%)
Arthritis	97 (89%)
Serositis	33 (30.3%)
Renal disorder	44 (40.4%)
Neurologic disorder	8 (7.3%)
Haematologic disorder	63 (57.8%)
Immunologic disorder	81 (74.3%)
Positive ANA	96 (88.1%)
Disease activity	
SLEDAI score (*n* = 99)	8 (4–14)
BILAG 2004, A or B in organ domain (*n* = 98)	
Constitutional	8 (8.2%)
Mucocutaneous	51 (52%)
Neuropsychiatric	10 (10.2%)
Musculoskeletal	51 (52%)
Cardiorespiratory	13 (13.3%)
Gastrointestinal	5 (5.1%)
Ophthalmic	5 (5.1%)
Renal	39 (39.8%)
Haematological	3 (3.1%)
Low C3/C4 (*n* = 99)	53 (53.5%)
Raised anti-dsDNA (*n* = 104)	43 (41.4%)
Autoantibodies (*n* = 104)	
ANA antibodies	99 (95.2%)
dsDNA antibodies	58 (55.8%)
U1RNP antibodies	34 (32.7%)
U3RNP antibodies	1 (1%)
SSa/Ro52 antibodies	20 (19.2%)
SSa/Ro60 antibodies	37 (35.6%)
SSb/La antibodies	6 (5.8%)
Medication	
Current oral corticosteroid use (*n* = 108)	94 (87%)
Usual oral corticosteroid dose (mg) (*n* = 75)	10 (7.5–20)
Current or previous antimalarial therapy	
Hydroxychloroquine	54 (49.5%)
Mepacrine	2 (1.8%)
Current or previous immunosuppressant exposure	
Azathioprine	9 (8.3%)
Methotrexate	5 (4.6%)
Mycophenolate mofetil	53 (48.6%)
Tacrolimus	1 (0.9%)
Cyclophosphamide	8 (7.3%)

Values are *n* (%) or median (IQR) as appropriate

### Deconvolution of whole blood RNA-seq data

**Fig. 1:**
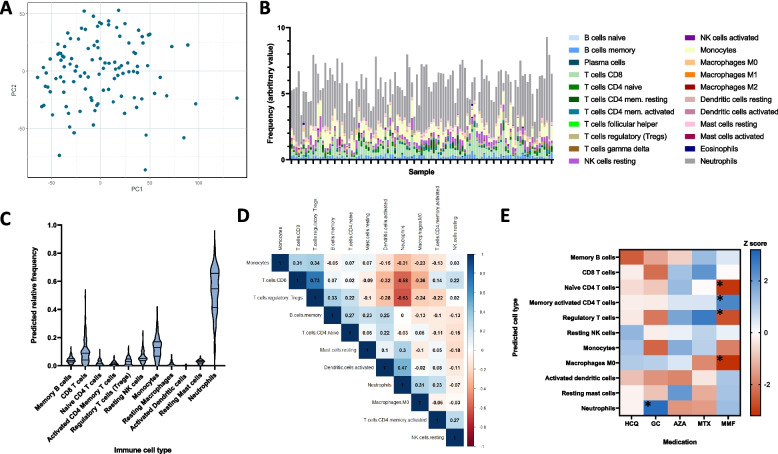
Predicted frequency of immune cell types in whole blood. **A** Principal component (PC) plot of PC1 and PC2 for all patient samples. **B** Relative frequency of each of the 22 cell types predicted using CIBERSORTx in each sample. **C** The predicted frequency of the 11 selected cell types in the whole cohort. **D** Correlation matrix of the 11 cell types in the whole cohort. Spearman *r* coefficients are shown and the intensity of the colour indicates the strength of the correlation. **E** Pseudoheatmap of the 11 cell types according to exposure to GC, HCQ and immunosuppressants. The colour shows the magnitude of the Z score, *p < 0.05. HCQ, hydroxychloroquine; GC, glucocorticoids; AZA, azathioprine; MTX, methotrexate; MMF, mycophenolate mofetil; CYC, cyclophosphamide

We analysed protein-coding genes in 110 patients. Principal component analysis did not identify any clear patient subgroups (Fig. 1A). Of 19,986 protein-coding genes, after removal of those with a raw count of zero in all samples, and those not matched during FPKM processing, there remained 17,231 for analysis. For further details of the workflow, see the Supplementary data file. For 11 of the cell types, frequencies could not be estimated in > 50% of samples, and therefore, we focussed on the remaining 11 cells: memory B cells, CD8 T cells, naïve CD4 T cells, memory activated CD4 T cells, regulatory T cells (Treg), resting NK cells, monocytes, M0 macrophages, activated dendritic cells, and resting mast cells (see Supplementary data). Of these, only monocytes and resting mast cells had a normal distribution. Neutrophils were the most abundant cell type (median [IQR] 54.7% [41.3–65.7] followed by monocytes (12.9% [6.80–17.0]). The distribution of the 11 cell types is shown in Fig. 1B and C.

The estimated absolute number of neutrophils was negatively correlated with Treg (*r* =  − 0.634, *p* < 0.0001), CD8 T cells (*r* =  − 0.560, *p* < 0.0001), and monocytes (*r* =  − 0.321, *p* = 0.0006) and positively correlated with activated dendritic cells (*r* = 0.413, *p* < 0.0001). Similarly, there was a strong correlation between Treg and CD8 T cells (*r* = 0.706, *p* < 0.0001) (Fig. 1D).

### Association between immune cell populations and disease phenotype

To assess whether organ involvement was associated with differing immune cell frequencies, patients with active disease (defined as a BILAG A or B score) in an organ domain were compared to those with inactive disease (C, D, or E score). There were no significant differences in immune cell frequency between active and inactive disease in any of the nine organ domains after correction for multiple testing (data not shown). In a sensitivity analysis in which active disease was defined as a BILAG A, B, or C score, again, no differences were observed. Similarly, there was no correlation between these immune cell frequencies and SLEDAI score or between patients with lower disease activity (SLEDAI < 10) and high disease activity (≥ 10) (data not shown).

### Association between immune cell populations and medication exposure

Most patients (94, 87%) were taking oral glucocorticoids (GCs) which was associated with increased predicted frequency of neutrophils compared to those not taking oral GCs (56.6% [44.1–66.8] vs 44.3% [33.4–50.3], *p* = 0.003) (Fig. 1E and Supplementary data S2). No statistically significant differences were observed in the other cell types between patients taking GC and those not taking GC. There were no differences in immune cell frequency in patients exposed (currently or previously) to azathioprine (AZA), methotrexate (MTX), hydroxychloroquine (HCQ), or cyclophosphamide (CYC).

**Fig. 2:**
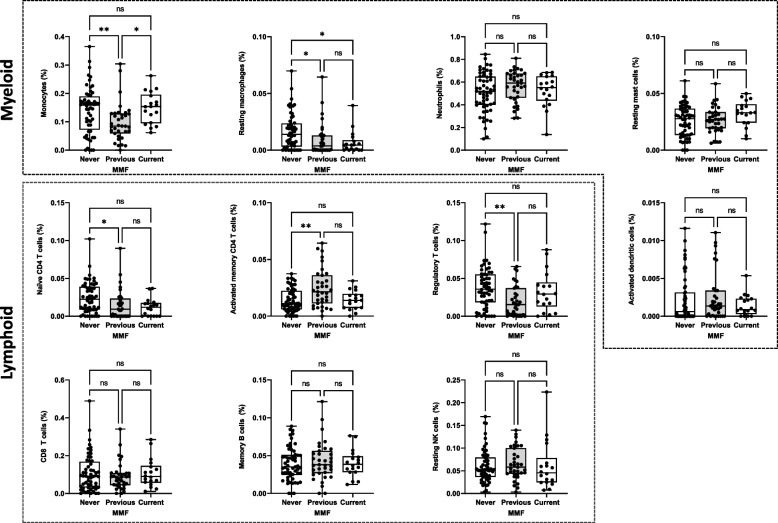
Differences in estimated cell proportions in patients receiving mycophenolate mofetil (MMF). Box plots show differences in estimated cell populations between patients never exposed, previously exposed, or currently receiving MMF. Horizontal line shows the median value and the box shows the IQR. Error bars show minimum and maximum values. Comparisons made using Kruskal–Wallis tests with Dunn’s correction for multiple comparisons. *p < 0.05, **p < 0.01

Compared to patients never exposed to mycophenolate mofetil (MMF), patients exposed to MMF had a significantly lower proportion of resting macrophages (0.44% [0–1.25] vs 1.39% [0.32–2.36], unadjusted *p* = 0.001), naïve CD4 T cells (0.96% [0–1.96] vs 2.25% [0.86–3.88], *p* = 0.002), and regulatory T cells (1.86% [0.29–3.93] vs 3.57% [1.84–5.52], *p* = 0.006), as well as a higher proportion of memory activated CD4 memory T cells (1.83% [0.87–2.78] vs 1.11% [0.60–2.23], *p* = 0.015). After Benjamini–Hochberg correction, these remained statistically significant (Supplementary file S3) and when MMF exposure was considered separately as current, previous, or never exposed, statistically significant differences remained for these four immune cell types (Fig. 2).

Patients exposed to MMF were more likely to have current or previous renal disease and were less likely to have a history of photosensitivity (Table 2). Similarly, patients with active mucocutaneous or musculoskeletal disease were less likely to have been prescribed MMF (57.9% never, 28.6% previous, 4.44% current, *p* = 0.023 and 61.4% never, 28.6% previous, 33.3% current, *p* = 0.004, respectively). Conversely, patients with active renal disease were more likely to be currently taking MMF (61.6% current, 25.7% ever, 33.3% never, *p* = 0.034). To exclude the effect of confounding by indication, multivariable ordered logistic regression analyses, using quartiles of cell frequency as the dependent variable, adjusted for age, gender, ethnicity, disease duration, renal disease, and corticosteroid use, were constructed which remained statistically significant for the 4 cell types above (Table 3).

**Table 2:** Clinical characteristics of patients related to exposure to MMF

	Never (*n* = 57)	Previous (*n* = 35)	Current (*n* = 18)	*p*
Age				
Sex (female)	54 (96.4%)	33 (94.3%)	17 (94.4%)	0.873
Ethnic background				0.408
White	34 (60.7%)	18 (54.6%)	10 (55.6%)	
Black	4 (7.14%)	6 (18.2%)	4 (22.2%)	
Asian	13 (21.2%)	4 (12.1%)	3 (16.7%)	
Other	5 (8.93%)	5 (15.2%)	1 (5.56%)	
Disease duration	18 (8, 17)	11 (7, 17)	8 (5, 13)	0.273
ACR criteria				
Malar rash	33 (58.9%)	17 (48.6%)	10 (55.6%)	0.626
Discoid rash	7 (12.5%)	5 (14.3%)	3 (16.7%)	0.900
** Photosensitivity**	**37 (66.1%)**	**15 (42.9%)**	**7 (38.9%)**	**0.035**
Oral ulcers	38 (67.9%)	18 (51.4%)	12 (66.7%)	0.266
Arthritis	49 (87.5%)	34 (97.1%)	14 (77.8%)	0.090
Serositis	18 (32.1%)	9 (25.7%)	6 (33.3%)	0.772
Neurological	4 (7.14%)	3 (8.57%)	1 (5.56%)	0.921
**Renal**	**21 (37.5%)**	**11 (31.4%)**	**12 (67.7%)**	**0.038**
Haematological	30 (53.6%)	22 (62.9%)	11 (61.1%)	0.651
Immunological	39 (69.6%)	27 (77.1%)	15 (83.3%)	0.460
ANA	49 (87.5%)	32 (91.4%)	15 (83.3%)	0.678
SLICC-DI	0 (0, 1)	0 (0, 2)	0 (0, 1)	0.862
Current prednisolone	46 (83.6%)	31 (88.6%)	17 (94.4%)	0.470
Daily prednisolone dose (mg/day)	10 (5–20)	10 (7.5–20)	10 (10–20)	0.280
SLEDAI score	10 (5.5–13.5)	6 (4–10)	10 (6–14)	0.051
Active disease (BILAG A/B)				
Constitutional (A or B)	6 (10.5%)	1 (2.86%)	1 (5.56%)	0.371
** Mucocutaneous (A or B)**	**33 (57.9%)**	**10 (28.6%)**	**8 (44.4%)**	**0.023**
Neuropsychiatric (A or B)	4 (7.02%)	4 (11.4%)	2 (11.1%)	0.735
** Musculoskeletal (A or B)**	**35 (61.4%)**	**10 (28.6%)**	**6 (33.3%)**	**0.004**
Cardiorespiratory (A or B)	8 (14.0%)	2 (5.71%)	3 (16.7%)	0.382
Gastrointestinal (A or B)	3 (5.26%)	0	2 (11.1%)	0.172
Ophthalmic (A or B)	2 (3.51%)	2 (5.71%)	1 (5.56%)	0.863
** Renal (A or B)**	**19 (33.3%)**	**9 (25.7%)**	**11 (61.1%)**	**0.034**
Haematological (A or B)	0	2 (5.71%)	1 (5.56%)	0.190
Low C3/C4	29 (51.2%)	11 (44.0%)	13 (72.2%)	0.173
Raised dsDNA	24 (42.9%)	12 (48.0%)	13 (72.2%)	0.094

**Table 3:** Ordered logistic regression models of the association between MMF exposure and quartiles of peripheral blood immune cell frequency

Cell type (quartiles)	Unadjusted			Model 1^a^			Model 2^b^		
	OR	95% CI	*p*	OR	95% CI	*p*	OR	95% CI	*p*
**Resting macrophages**	**0.354**	**0.176, 0.771**	**0.004**	**0.386**	**0.178, 0.840**	**0.016**	**0.329**	**0.177, 0.872**	**0.022**
**Naïve CD4 + T cells**	**0.390**	**0.195, 0.776**	**0.007**	**0.398**	**0.184, 0.859**	**0.019**	**0.369**	**0.167, 0.816**	**0.014**
**Regulatory T cells**	**0.363**	**0.181, 0.728**	**0.004**	**0.227**	**0.099, 0.521**	** < 0.001**	**0.216**	**0.092, 0.508**	** < 0.001**
**Activated CD4 + memory T cells**	**1.952**	**0.984, 3.869**	**0.055**	**2.174**	**1.014, 1.659**	**0.046**	**2.447**	**1.120, 5.346**	** < 0.001**
Monocytes	0.528	0.266, 1.045	0.067	0.527	0.244, 1.139	0.103	0.579	0.262, 1.279	0.176

^a^Model 1: adjusted for age, gender, ethnicity, and disease duration

^b^Model 2: as for model 1 plus presence of renal disease (BILAG A/B) and corticosteroid use (Y/N)

### Transcriptomic signature associated with MMF exposure

**Fig. 3:**
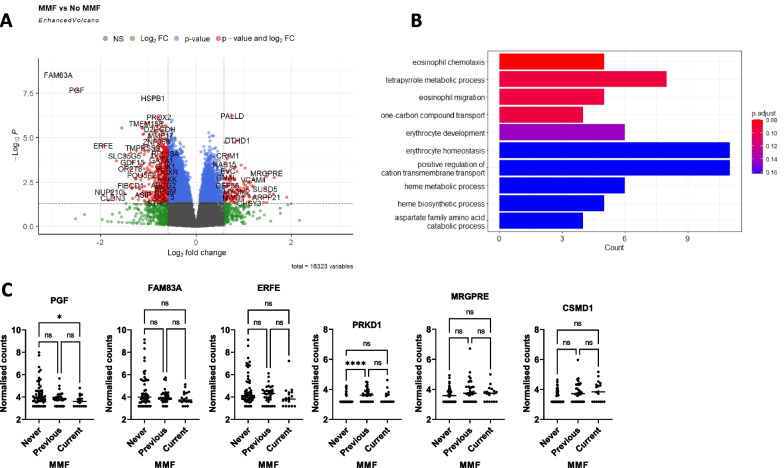
Whole blood gene signature in patients exposed to MMF. **A** Volcano plot to show differentially expressed genes between patients exposed to MMF or not. The *y*-axis shows -log10 adjusted *p* value and *x*-axis shows log2 fold change. Genes in red are differentially upregulated or downregulated with log2 fold change > 0.5 and adjusted *p* value < 0.05. **B** Gene set enrichment analysis of the 2607 differentially expressed genes. The *x*-axis shows the number of genes contributing to the term and the colour of the bar represents the *p* value. **C** Box plots of the 3 genes most significantly upregulated or downregulated according to MMF exposure. Horizontal bar shows median. Comparisons with Kruskal–Wallis test with Dunn’s correction. *p < 0.05, ****p < 0.0001

There were 2607 differentially expressed genes (DEGs) between patients who were exposed (current or ever) to MMF compared to those who were not (log2 fold change of 0.5 and adjusted *p* value of < 0.05). Of these, 767 were upregulated, and 1840 were downregulated (Fig. 3A). Gene set enrichment analysis (GSEA) of DEGs identified over-representation of genes sets related to erythrocyte development and eosinophil migration (Fig. 3B). The genes upregulated or downregulated with smallest adjusted *p*-value were further examined (3 of each). Of the 3 downregulated DEGs, expression of *PGF* was statistically significantly lower in patients currently taking or ever exposed to MMF. Similarly, of the 3 upregulated DEGS, *CSMD1* was increased in patients currently taking or ever exposed to MMF (Fig. 3C).

**Fig. 4:**
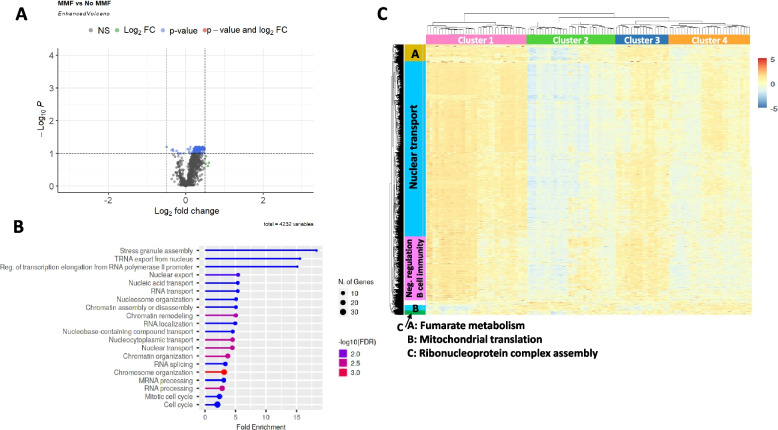
Predicted gene expression in CD4 + T cells in patients exposed to MMF. **A** Volcano plot of DEGs predicted in the CD4 T cell subset according to exposure to MMF. Horizontal line shows -log10 adjusted *p* value and vertical line shows log2 fold change. **B** GO analysis of over-represented biological pathways in the 157 DEGs with adjusted *p* < 0.05 between patients exposed or not to MMF. **C** Heatmap of genes in CD4 T cells with hierarchical clustering of samples (horizontal) and genes (vertical). The vertical bars show the top GO biological process for each cluster of genes

As MMF exposure was associated with changes to T cell frequency, gene expression within the CD4 cell subset was predicted using CIBERSORTx high resolution mode. Of 4232 genes, only 157 were differentially expressed (adjusted *p* < 0.05) according to exposure to MMF (Fig. 4A). There were insufficient genes to perform GSEA, but gene ontology (GO) analysis of biological function identified over-representation of genes involved in nucleocytoplasmic transport (Fig. 4B). Hierarchical clustering of CD4 T cell transcripts identified 4 patients clusters (cluster 1; 42 patients, cluster 2; 30 patients, cluster 30; 14 patients, cluster4; 21 patients) (Fig. 4C) and 5 gene clusters with identifiable pathway enrichment. GO analysis of the gene clusters identified pathways related to nuclear transport (fold enrichment 2.67, enrichment false discovery rate [FDR] 9.97 × 10^−10^), negative regulation of B cell immunity (fold enrichment 11.9, enrichment FDR 0.013), and fumarate metabolism (fold enrichment 73.5, enrichment FDR 0.030). The pathway genes for each of the top 3 GO terms in each of the 5 gene clusters are shown in the Supplementary Table S4. The characteristics of patients in each of the 4 clusters is shown in Table 4. Patients in clusters 2 and 4 were older and were more likely to be receiving prednisolone and less likely to be exposed to methotrexate. Overall, the expression of most transcripts was higher in clusters 2 and 4 compared to clusters 1 and 3, except for a small group of transcripts which were enriched with genes related to mitochondrial translation (GO: 0070125). There was no difference in lupus disease activity by BILAG organ system, exposure to MTX or AZA, between the clusters. Patients in clusters 2 and 3 were more likely to have a history of mucosal ulceration.

**Table 4:** Characteristics of patients in each CD4 T cells transcriptional cluster

	Cluster 1 *N* = 42	Cluster 2 *N* = 30	Cluster 3 *N* = 14	Cluster 4 *N* = 24	*p*
**Age (years)**	**35 (27–46)**	**41 (34–53)**	**34 (19–38)**	**42 (34–52)**	**0.027**
Gender (female)	30 (95.1%)	29 (96.7%)	14 (100%)	22 (91.7%)	0.790
Ethnicity					
White	27 (65.9%)	17 (56.7%)	6 (46.2%)	11 (50.0%)	0.396
Black	5 (11.9%)	5 (16.7%)	3 (23.1%)	1 (4.55%)	
Asian	6 (14.3%)	4 (13.3%)	4 (30.8%)	6 (27.3%)	
Other	3 (7.14%)	4 (13.3%)	0	4 (18.2%)	
Disease duration (years)	8 (6–15)	11 (9–17)	8.5 (6–11)	11.5 (8–20.5)	0.228
SLICC DI	0 (0–1)	0 (0–1)	0 (0–0.5)	0 (0–1)	0.561
ACR criteria					
Malar rash	21 (51.2%)	14 (46.7%)	9 (64.3%)	16 (66.7%)	0.409
Discoid rash	9 (22.0%)	1 (3.33%)	1 (7.14%)	4 (16.7%)	0.124
Photosensitivity	21 (51.2%)	15 (50.0%)	9 (64.3%)	14 (58.3%)	0.777
**Oral ulcers**	**24 (58.5%)**	**23 (76.7%)**	**11 (78.6%)**	**10 (41.7%)**	**0.032**
Arthritis	38 (92.7%)	26 (86.7%)	12 (85.7%)	21 (87.5%)	0.815
Serositis	14 (34.2%)	7 (23.3%)	4(28.6%)	8 (33.3%)	0.777
Neurological	3 (7.32%)	2 (6.67%)	1 (7.14%)	2 (8.33%)	0.997
Renal	15 (36.6%)	14 (46.7%)	7 (50.0%)	8 (33.3%)	0.621
Haematological	22 (52.7%)	14 (46.7%)	9 (64.3%)	18 (75.0%)	0.174
Immunological	29 (70.7%)	24 (80.0%)	12 (85.7%)	16 (66.7%)	0.480
ANA	37 (90.2%)	25 (85.3%)	12 (85.7%)	22 (91.7%)	0.754
Disease activity					
SLEDAI score	8 (4–2)	8 (4–12)	12 (8–18)	6 (4–12)	0.145
Active disease (BILAG A/B)					
Constitutional	4 (9.76%)	3 (10.0%)	1 (7.14%)	0	0.463
Mucocutaneous	20 (48.8%)	15 (50.0%)	6 (42.9%)	10 (41.7%)	0.912
Neuropsychiatric	4 (9.76%)	3 (10.0%)	1 (7.14%)	2 (8.33%)	0.988
Musculoskeletal	21 (5.12%)	17 (56.7%)	7 (50.0%)	6 (25.0%)	0.105
Cardiorespiratory	4 (9.76%)	4 (13.3%)	0	5 (20.83%)	0.267
Gastrointestinal	2 (4.88%)	1 (33.3%)	0	2 (8.33%)	0.669
Ophthalmic	1 (2.44%)	2 (6.67%)	0	2 (8.33%)	0.538
Renal	13 (31.7%)	12 (40.0%)	6 (42.9%)	8 (33.3%)	0.826
Haematological	1 (2.44%)	2 (6.67%)	0	0	0.422
Medication					
**Oral prednisolone**	**32 (78.1%)**	**29 (96.7%)**	**10 (76.9%)**	**23 (95.8%)**	**0.042**
Oral prednisolone dose (mg/day)	10 (5–20)	10 (7.5–20)	10 (5–15)	12.5 (10–20)	0.495
MMF exposed	23 (56.1%)	13 (43.3%)	4 (28.6%)	13 (54.2%)	0.284
MMF current	8 (19.5%)	5 (16.7%)	3 (21.4%)	2 (8.33%)	0.642
AZA exposed	5 (12.2%)	2 (6.67%)	1 (7.14%)	1 (4.17%)	0.684
AZA current	3 (7.32%)	2 (6.67%)	1 (7.14%)	0	0.613
** MTX exposed**	**1 (2.44%)**	**0**	**4 (28.7%)**	**0**	** < 0.001**
** MTX current**	**1 (2.44%)**	**0**	**3 (21.4%)**	**0**	**0.002**

As MMF exposure was associated with fewer predicted resting macrophages, predicted gene expression in monocytes was also investigated in high resolution mode. There were no differentially expressed genes in patients exposed or not exposed to MMF with adjusted *p* < 0.05. Cluster analysis suggested 7 patient groups and 9 gene clusters (see Supplementary Figure S2).

## Discussion

We used CIBERSORTx to predict the immune cell composition in the blood of patients with active SLE. In the interpretation of deconvoluted transcriptomic data, it is important to recognise that, whilst this may not be directly comparable to studies using flow cytometric methods, CIBERSORT has been validated against flow cytometry data in human blood [20]. Of the 22 cell types, 11 were reliably identified in over 50% of patients and used for further analysis. In our data, there were notable correlations between the predicted numbers of neutrophils, Treg, monocytes, and CD8 + T cells, which have not been observed in other clinical contexts [6, 21] suggesting that these finding may be disease- or medication-specific.

In this study, we did not find any associations between predicted immune cell frequency and active disease in any of the nine BILAG-2004 domains. This contrasts with a small flow cytometry study which identified increased frequency of T cells and reduced B cell and NK cell frequency in patients with active glomerulonephritis (lupus nephritis class III or IV) [22]. Absolute counts of NK cells were also decreased in patients with renal involvement. This discrepancy between the studies could be attributed to using BILAG A/B scores, rather than a histological definition of nephritis, which is likely to encompass a wider group of patients, or to important differences between flow cytometry and transcriptomic analyses. Our findings in this study suggest that the principal immune cell composition in the peripheral blood of patients with SLE does not relate to specific organ activity.

Oral GCs were noted to be associated with a significant increase in the proportion of neutrophils in the peripheral blood, consistent with established literature validating our approach [23]. Of the remaining immunosuppressants assessed in this study, MMF use associated with statistically significant differences in the predicted frequency of immune cells (macrophages, memory activated CD4 T cells, naïve CD4 T cells and regulatory T cells). Although patients currently or previously treated with MMF were more likely to have renal disease and less likely to have musculoskeletal or mucocutaneous disease, in multivariable models, the relative frequency of these 4 immune cell populations was independently associated with MMF exposure.

Recently a large study by Northcott et. al. (2022) investigated gene module expression in 210 patients with SLE using a commercially available assay [24]. In this study, current MMF use was associated with a significantly reduced plasmablast signature. Similarly, AZA use was associated with a lower B cell signature and higher IFN signature. The number of patients exposed to AZA in our study was small, and so it was likely underpowered to identify differences in gene expression related to AZA use. Patients treated with prednisolone had lower pDC, B cell, T cell, and plasmablast signatures, and supporting our findings, higher doses of prednisolone (> 7.5 mg/day) was associated with higher expression of neutrophil-related genes, which may reflect an absolute increase in neutrophil number in the circulation.

A previous small study examined the effects of MMF and cyclophosphamide treatment on peripheral blood lymphocytes and NK cells after 4 weeks using flow cytometry [25]. It identified a significant increase in CD3^+^CD4^+^ Th cells over the baseline (pre-treatment) level when taking either MMF or CYC. Whilst CIBERSORTx identified higher memory activated CD4 T cells with MMF use in our study, which would be consistent with more CD4 Th cells, there were also fewer naïve CD4 T cells. This could be because our study analysed multiple subgroups of CD4 T cells, rather than CD4 Th cells as a single group. A similar study reported that the frequencies and absolute numbers of CD27-IgD + CD38 +  + transitional and CD27-IgD + CD38 + naïve B cells, absolute numbers of CD27 + IgD + pre-switched memory B cells, and B cell counts overall were lower in patients taking AZA compared to both MMF-treated patients or patients not taking immunosuppressive therapy [26]. Conversely, patients taking MMF had significantly lower frequencies and counts of antibody-producing cells than patients taking AZA or no immunosuppressants. In our study, plasma cells were not predicted to be present in the peripheral blood for many patients. This is in contrast to the study by Northcott et. al. [24] in which a plasmablast/plasma cell signature was detected. This is likely due to differences in the gene panel between the commercially available assay and the LM22 dataset. Furthermore, the plasmablast signature was lower in patients receiving higher doses of prednisolone (defined by Northcott as > 7.5 mg/day). In their study, 15% of patients were receiving this > 7.5 mg/day, compared to 50% in our cohort which may have suppressed the plasma cell signature further.

We did not identify any significant differences in cell frequency in patients receiving either CYC or AZA although the number of patients in each of these groups was low in our study. A study focusing on juvenile-onset SLE in 2020 identified four patient groups based on eight immune cell subsets and defined predominantly by differences in the frequency of CD4 and CD8 T cells. Interestingly, there were significant differences in MMF use, but not other immunosuppressants, between the groups [27].

In our study, changes in predicted immune cell frequency were observed according to current/previous exposure to MMF rather than whether patients were currently receiving MMF. We were unable to determine whether, in those patients previously exposed, MMF withdrawal was recent enough to still affect cell proportions. In patients with SLE, others have demonstrated prolonged changes in the B cell compartment following withdrawal of MMF [28].

Although there were a significant number of DEGs related to MMF exposure in whole blood, only a small number of DEGs were identified within the CD4 T cell subset. This suggests that the transcriptomic difference observed in whole blood may be driven by changes in immune cell frequency, rather than significant differences in the transcriptome within an individual cell type. Further studies should confirm this observation using sorted cells. Within the CD4 T cell population, the patient clusters differed by age, previous mucosal ulceration, prednisolone use, and MTX exposure. Genes related to nuclear transport including *NUP88* and *NUP105* appeared to be expressed at lower levels in clusters 2 and 4, which comprised older patients, consistent with reports of reduced nuclear transport protein expression in ageing [29].

An important limitation of our study is that the number of patients currently immunosuppressants other than MMF was relatively small, which may be because patients were recruited just prior to an escalation in therapy (mostly commonly starting rituximab). Furthermore, in our study, low estimated cell numbers were obtained for 11/22 cells in the LM22 matrix. This included plasma cells which have been reported to be increased in the peripheral blood in active SLE patients [30] along with other potentially relevant cell types including naïve B cells and activated macrophages. As our data does not include a healthy control population, we were unable to directly compare SLE with heathy controls, but for the 11 cell types we analysed, their estimated frequencies were similar to those reported by others [3]. Our study was cross-sectional, and so we were unable to study whether these cell populations were stable over time or how they might change in response to drug treatment. Finally, whilst estimations of cell populations based on transcriptomic profile have been shown to correlate with flow cytometry findings in other contexts, this methodology has not been validated in patients with SLE.

## Conclusion

Drug therapy is associated with important differences in the whole blood transcriptomic signature in patients with SLE which may persist even after the medication is withdrawn. A better understanding of the short- and medium-term consequences of these changes and how they relate to prognosis in SLE is needed. Persistent immune system changes could influence refractoriness to future treatments. Importantly, our results demonstrate the need to adequately adjust for background medication use, especially in studies using whole blood transcriptomics.

### Supplementary Information

**Additional file 1.** 

## Data Availability

The data underlying this article are available on reasonable request to the corresponding author dependent upon the nature of the request, the availability of the data, and its intended use.

## Abbreviations

AZA: Azathioprine
BILAG: British Isles Lupus Assessment Group
CYC: Cyclophosphamide
DEG: Differentially expressed gene
FDR: False discovery rate
FPKM: Fragments per kilobase million
GC: Glucocorticoids
GSEA: Gene set enrichment analysis
HCQ: Hydroxychloroquine
IFN: Interferon
MMF: Mycophenolate mofetil
MTX: Methotrexate
NK: Natural killer
RNA-seq: Ribonucleic acid sequencing
SLE: Systemic lupus erythematosus
SLEDAI: Systemic lupus erythematosus disease activity index
SLICC: Systemic lupus international collaborating clinics
