# Correction: Deconvolution of whole blood transcriptomics identifies changes in immune cell composition in patients with systemic lupus erythematosus (SLE) treated with mycophenolate mofetil

**DOI:** 10.1186/s13075-023-03160-1

**Published:** 2023-09-04

**Authors:** Mumina Akthar, Nisha Nair, Lucy M. Carter, Edward M. Vital, Emily Sutton, Neil McHugh, British Isles Lupus Assessment Group Biologics Register (BILAG BR) Consortium, MASTERPLANS Consortium, Ian N. Bruce, John A. Reynolds

**Affiliations:** 1 Rheumatology Department, Sandwell and West Birmingham NHS Trust, Birmingham, UK; 2 Centre for Genetics and Genomics Versus Arthritis, Centrefor Musculoskeletal Research, Manchester Academic Health Science Centre, The University of Manchester, Manchester, UK; 3 Leeds Institute of Rheumatic and Musculoskeletal Medicine, University of Leeds, Leeds, UK; 4 NIHR Leeds Biomedical Research Centre, Leeds Teaching Hospitals NHS Trust, Leeds, UK; 5 Centre for Epidemiology Versus Arthritis, Division of Musculoskeletal & Dermatological Sciences, The University of Manchester, Manchester, UK; 6 Department of Pharmacy and Pharmacology, University of Bath, Bath, UK; 7 NIHR Manchester Biomedical Research Centre, Manchester University Hospitals NHS Foundation Trust, Manchester Academic Health Science Centre, Manchester, UK; 8 Rheumatology Research Group, Institute of Inflammation and Ageing, College of Medical and Dental Sciences, University of Birmingham, Birmingham, UK

**Correction: Arthritis Res Ther 25, 111 (2023)**

**https://doi.org/10.1186/s13075-023-03089-5**

Following publication of the original article [1], the authors identified an error to the last name of Mumina Akthar.

The incorrect author name is: Mumina Akhtar

The correct author name is: Mumina Akthar

The author group has been updated above and the original article [1] has been corrected.
