# Supplementary material for: Deconvolution of whole blood transcriptomics identifies changes in immune cell composition in patients with systemic lupus erythematosus (SLE) treated with mycophenolate mofetil
Source: Arthritis Res Ther. 2023 Jun 30;25:111. doi: 10.1186/s13075-023-03089-5 (PMC10311871; doi:10.1186/s13075-023-03089-5)

Supplementary methods

*Deconvolution of whole blood RNASeq data*

We conducted FPKM-processing on the protein-coding genes identified from RNA sequencing and found 6.35% of genes were unmatched. We then removed those genes along with those with undetectable expression in all samples leaving 17229 genes for analysis (dataset 1). We also filtered the protein-coding genes and removed the genes with undetectable expression in all samples. Following FPKM-processing, 4.12% of genes were unmatched and were removed along with any further genes with undetectable expression in all samples leaving 17231 genes for analysis.

As both methods led to a very similar number of genes, both FPKM files were individually uploaded onto CIBERSORTx and cell deconvolution using the LM22 signature matrix provided by CIBERSORTx was performed.

Computational cell type quantification methods can be classified into two categories: marker gene-based approaches and deconvolution-based approaches as shown in the paper by Sturm et al. CIBERSORT is deconvolution-based. It uses support vector regression in combination with the knowledge of expression profiles in a signature matrix to accurately estimate the relative immune proportions of cells from bulk tissue transcriptomes. CIBERSORTx is the next generation version of CIBERSORT with additional features such as supporting single-cell RNA-seq data. We therefore proceeded to use CIBERSORTx and its webtool for our study.

LM22 is signature matrix provided by CIBERSORTx that distinguishes 22 cell types. These are: naïve B cells, memory B cells, plasma cells, CD8 T cells, naïve CD4 T cells, memory resting CD4 T cells, memory activated CD4 T cells, follicular helper T cells, regulatory T cells, gamma delta T cells, resting NK cells, activated NK cells, monocytes, macrophages M0, macrophages M1, macrophages M2, resting dendritic cells, activated dendritic cells, resting mast cells, activated mast cells, eosinophils and neutrophils. Affymetrix HGU133A microarray data was utilised for the creation of LM22.

The output from CIBERSORTx for both the whole dataset and protein-coding genes only were then directly correlated for each cell subtype using IBM® SPSS® version 26. The correlation coefficient was 1.000 for all 22 cell types. Further analysis was conducted using the protein-coding dataset as this had fewer unmatched genes.

Supplementary tables

Table S1: Predicted cell frequencies in the whole cohort

| Cell type | Patients with predicted frequency of zero | Median relative frequency (whole cohort) | IQR |
| --- | --- | --- | --- |
| NK cells, resting | 0% | 5.19% | 3.67, 8.25 |
| Neutrophils | 0% | 54.7% | 41.3, 65.7 |
| Mast cells, resting | 3% | 2.84% | 1.72, 3.67 |
| B cells, memory | 4% | 3.69% | 2.69, 5.23 |
| T cells, CD8 | 5% | 8.88% | 4.02, 13.40 |
| T cells, CD4, memory activated | 5% | 1.40% | 0.75, 2.47 |
| Monocytes | 5% | 12.9% | 6.80, 17.0 |
| T cells, regulatory | 13% | 2.90% | 0.67, 4.82 |
| T cells, CD4 naive | 24% | 1.43% | 0.09, 3.30 |
| Macrophages, M0 | 27% | 0.66% | 0.00, 1.93 |
| Dendritic cells, activated | 34% | 0.10% | 0.00, 0.27 |
| T cells CD4, memory resting | 59% | 0.00% | 0.00, 1.05 |
| T cells, gamma-delta | 64% | 0.00% | 0.00, 0.91 |
| NK cells, activated | 68% | 0.00% | 0.00, 0.24 |
| Plasma cells | 76% | 0.00% | 0.00, 0.00 |
| Macrophages, M2 | 83% | 0.00% | 0.00, 0.00 |
| B cells, naive | 85% | 0.00% | 0.00, 0.00 |
| T cells, follicular helper | 91% | 0.00% | 0.00, 0.00 |
| Dendritic cells, resting | 91% | 0.00% | 0.00, 0.00 |
| Macrophages, M1 | 92% | 0.00% | 0.00, 0.00 |
| Eosinophils | 92% | 0.00% | 0.00, 0.00 |
| Mast cells, activated | 98% | 0.00% | 0.00, 0.00 |

Table S2: Predicted cell frequencies in patients concurrently taking oral prednisolone

| **Cell type** | **Yes** | **No** | **p-value** | **Adjusted p-value*** |
| --- | --- | --- | --- | --- |
| Neutrophils | 56.6 | 44.3 | 0.003 | 0.033 |
| Monocytes | 11.7 | 16.4 | 0.017 | 0.092 |
| CD8 T cells | 8.50 | 11.7 | 0.025 | 0.092 |
| Regulatory T cells | 2.57 | 4.37 | 0.036 | 0.098 |
| Activated dendritic cells | 0.11 | 0.03 | 0.096 | 0.211 |
| Memory B cells | 3.67 | 4.87 | 0.164 | 0.301 |
| Resting mast cells | 2.74 | 3.08 | 0.351 | 0.466 |
| Macrophages M0 | 0.71 | 0.47 | 0.351 | 0.466 |
| Naïve CD4 T cells | 1.45 | 2.24 | 0.382 | 0.466 |
| Memory activated CD4 T cells | 1.39 | 1.52 | 0.749 | 0.784 |
| Resting NK cells | 5.18 | 5.32 | 0.784 | 0.784 |

Table shows media values and p-value following Mann-Whitney U test

*after Benjamini-Hochberg correction

Table S3: Predicted cell frequencies in patients exposed to MMF

| **Cell type** | **Yes** | **No** | **p-value** | **Adjusted p-value*** |
| --- | --- | --- | --- | --- |
| Neutrophils | 57.0 | 51.8 | 0.194 | 0.356 |
| Monocytes | 11.4 | 15.8 | 0.050 | 0.111 |
| CD8 T cells | 8.64 | 9.09 | 0.940 | 0.940 |
| Regulatory T cells | 1.86 | 3.58 | 0.006 | 0.024 |
| Activated dendritic cells | 0.19 | 0.06 | 0.265 | 0.364 |
| Memory B cells | 3.80 | 3.57 | 0.652 | 0.796 |
| Resting mast cells | 2.87 | 2.78 | 0.242 | 0.364 |
| Macrophages M0 | 0.43 | 1.39 | 0.001 | 0.009 |
| Naïve CD4 T cells | 0.96 | 2.26 | 0.002 | 0.009 |
| Memory activated CD4 T cells | 1.83 | 1.12 | 0.015 | 0.041 |
| Resting NK cells | 5.48 | 5.19 | 0.779 | 0.857 |

Table shows media values and p-value following Mann-Whitney U test

*after Benjamini-Hochberg correction

Table S4: Predicted gene clusters in CD4 T cells for each of the top 3 GO biological pathways

| Cluster number | Key GO biological pathways | Genes |
| --- | --- | --- |
| 1 | Fumerate metabolic processing (GO: 0006106) | GOT1 GOT2 |
|  | SRP-dependent cotranslational protein targeting to membrane (GO: 0006614) | RPL3 RPL22 RPL23 RPS3 RPL30 SRP68 RPL4 |
|  | Protein targeting to ER (GO: 0045047) | RPL3 RPL22 RPL23 RPS3 RPL30 SRP68 RPL4 |
| 2 | Nuclear export (GO: 0051168) | NUP160 RANBP3 NSUN2 LSG1 NDC1 U2AF2 NUP133 SMG6 SEH1L ALKBH5 NUP88 SUPT6H CPSF6 SRSF3 HSPA9 NUP155 NCBP2 SRSF11 KHDRBS1 HNRNPA2B1 DDX39A SRSF6 CSE1L XPO5 PCID2 XPO4 SRRM1 HNRNPA1 LTV1 DHX9 SRSF1 WDR33 CTDSPL2 NUP54 DHX38 TP53 POLR2D EIF4E BAG3 NUP205 SRSF2 NUP35 ABCE1 PHAX NEMF TSC1 NMD3 MALT1 RRS1 NPM1 XPOT POM121 NUP62CL STYX SDAD1 RBM15B POM121C DUSP16 |
|  | RNA localization (GO: 006403) | NUP160 NSUN2 NDC1 U2AF2 NUP133 SMG6 SIDT1 SEH1L KHSRP ALKBH5 NUP88 SUPT6H CPSF6 SRSF3 NUP155 NCBP2 SRSF11 ZNHIT6 TCP1 KHDRBS1 HNRNPA2B1 DDX39A SRSF6 XPO5 PCID2 DKC1 SRRM1 HNRNPA1 DHX9 SRSF1 WDR33 LRPPRC NUP54 HNRNPA1L2 DHX38 POLR2D SHQ1 NAF1 CCT6A ATM EIF4E NUP205 CCT8 SRSF2 NUP35 PHAX TSC1 CCT2 SNUPN HNRNPA3 EXOSC10 TOMM20 ATR CKAP5 NPM1 XPOT POM121 NUP62CL ATXN2 RBM15B POM121C PARN |
|  | NcRNA processing (GO: 0034470) | ELAC2 UTP18 ZCCHC8 NSUN2 USP36 WDR3 TRMT11 THUMPD1 RRP15 ELP1 TRNT1 DDX1 WBP11 DIMT1 DDX18 ESF1 PUS7 ALKBH1 CSTF2 INTS9 INTS10 GTPBP4 INTS2 PUS3 KRR1 DROSHA BRIX1 THADA ZNHIT6 NSUN4 UTP25 HEATR1 FCF1 RCL1 WDR55 UTP20 TENT4B TRMT13 HNRNPA2B1 METTL8 MOCS3 MPHOSPH10 RIOK1 SBDS TRMT5 HELB OSGEPL1 NGDN DKC1 NOL11 UTP3 ELP3 THUMPD3 TUT4 WDR36 MTO1 MPHOSPH6 C2orf49 TEX10 ALKBH8 WDR12 INTS14 NOB1 URB1 SHQ1 NAF1 CDKAL1 RPL7L1 RPL7 UTP23 INTS4 QTRT2 TSEN2 NIFK RRP1B TYW3 TFB2M RPP14 WDR43 UTP15 NSA2 INTS6L RPUSD4 BMS1 METTL3 NOLC1 SMAD3 TSR1 ZCCHC4 DTWD2 TRMT61B FAM98B EXOSC1 RSL1D1 EXOSC10 TP53RK RPL15 DDX10 RRS1 PRKRA BCDIN3D TRMT2B NOL8 MAK16 YTHDF2 TSEN15 TYW1 NSUN6 FDXACB1 RPL17 PARN TYW1B DDX52 DUS4L MDN1 |
| 3 | Negative regulation of B cell mediated immunity (GO: 0002713) | PARP3 FOXP3 C4BPB FOXJ1 SUSD4 |
|  | Negative regulation of immunoglobulin mediated immune response (GO: 0002890) | PARP3 FOXP3 C4BPB FOXJ1 SUSD4 |
|  | Ribosomal large subunit assembly (GO: 0000027) | NLE1 RPL6 RPL5 PPAN RPL11 RPL10 DDX28 RPL23A |
| 4 | Mitochondrial translational elongation (GO: 0070125) | MRPS26 MRPS5 MRPL58 TUFM MRPS21 |
|  | Mitochondrial translation (GO: 0032543) | MRPS26 MRPS5 MRPL58 TUFM MRPS21 |
|  | Translational elongation (GO: 0006414) | MRPS26 MRPS5 MRPL58 TUFM MRPS21 |
| 5 | Ribonuceloprotein complex assembly (GO: 0022618) | PRPF19 DHX30 TARBP2 GEMIN4 SF3A3 |
|  | Ribonuceloprotein complex subunit organisation (GO: 0071826) | PRPF19 DHX30 TARBP2 GEMIN4 SF3A3 |
|  | Ribonuceloprotein complex biogenesis (GO: 0022613) | PRPF19 DDX54 DHX30 IMP4 TARBP2 GEMIN4 SF3A3 |

Supplementary figures:

**Figure S1: Predicted absolute cell numbers in each patient sample.**

The graph shows the predicted absolute cell numbers across 22 cell types in each sample.

**Figure S2: Cell-specific gene expression in monocytes**

A. Volcano plot of 4535 genes in patients exposed to MMF compared to not exposed. The horizontal line shows an adjusted P value of 0.1. B. Table of the 7 genes with adjusted P value <0.1. C. Heatmap of the 4535 with hierarchal clustering by patient (columns) and genes (rows). A total of 7 patient clusters were identified and 9 gene clusters. The colour shows relative expression level scaled for each gene. The principal GO biological term is shown for each of the 9 clusters.

Figure S1: Estimated absolute cell counts across all samples
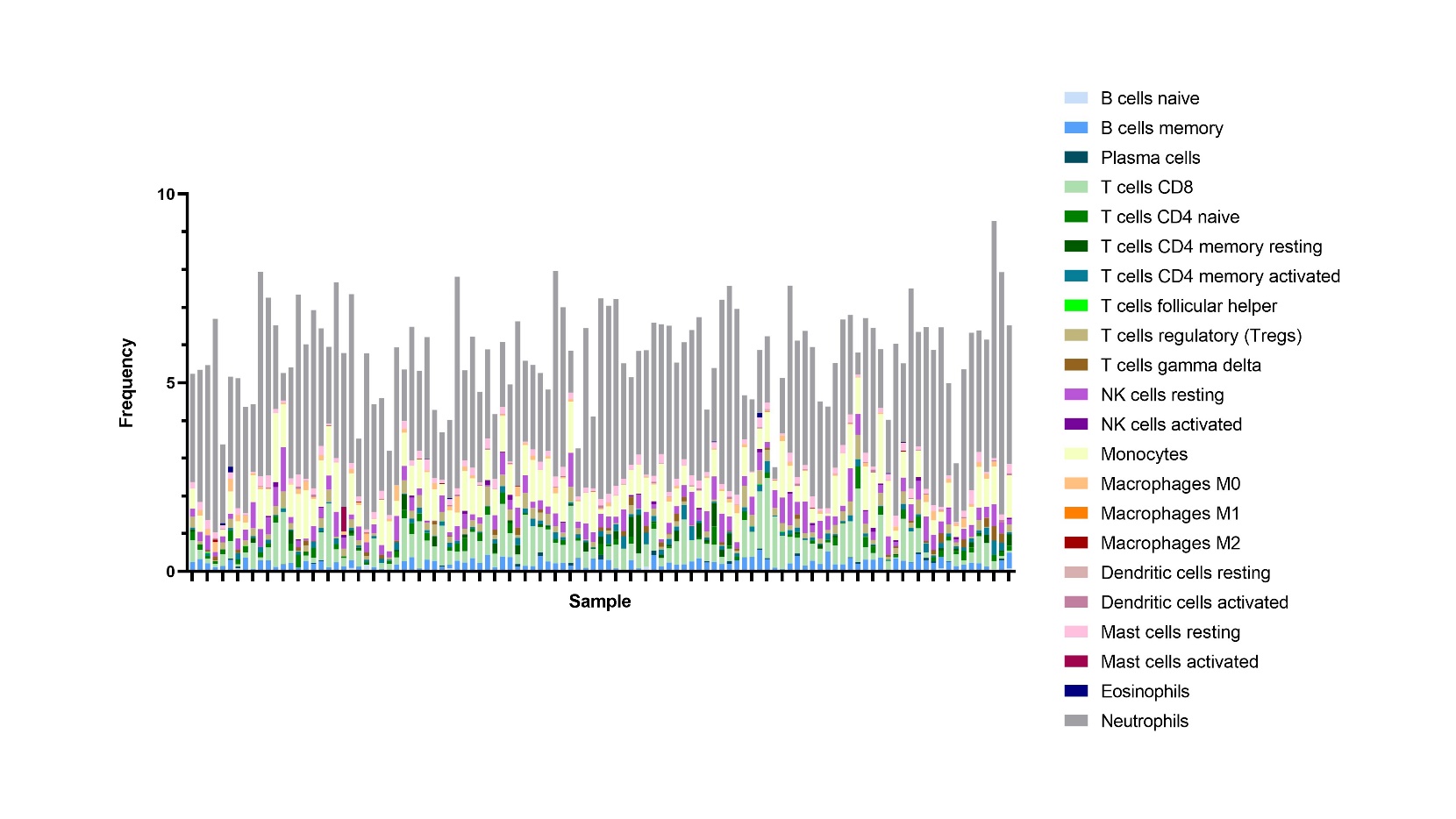


Figure S2:


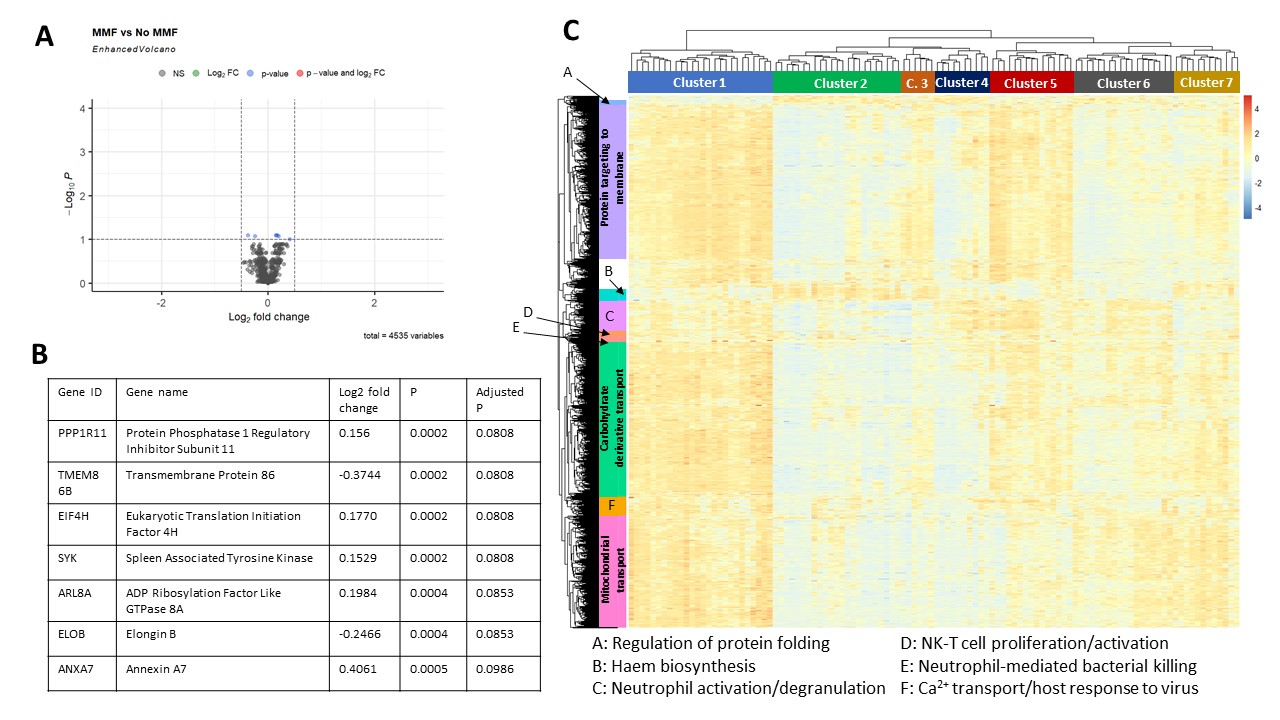

Supplement: Supplementary file 1 — Additional file 1. [file 13075_2023_3089_MOESM1_ESM.docx]
